# Species Specificity of the Putative Male Antennal Aphrodisiac Pheromone in* Leptopilina heterotoma*,* Leptopilina boulardi*, and* Leptopilina victoriae*

**DOI:** 10.1155/2015/202965

**Published:** 2015-12-29

**Authors:** Ingmar Weiss, Joachim Ruther, Johannes Stökl

**Affiliations:** ^1^Institute for Zoology, University of Regensburg, Universitätsstraße 31, 93053 Regensburg, Germany; ^2^Institute of Biology, Free University of Berlin, Haderslebener Straße 9, 12163 Berlin, Germany

## Abstract

Male antennal aphrodisiac pheromones have been suggested to elicit female receptiveness in several parasitic Hymenoptera, including* Leptopilina boulardi*. None of the proposed pheromones, however, has been fully identified to date. It is also unknown whether these antennal pheromones are species specific, because the species specificity of mate recognition and courtship elicitation in* Leptopilina* prevented such experiments. In this study we present an experimental design that allows the investigation of the species specificity of the putative male aphrodisiac pheromone of* L. heterotoma, L. boulardi,* and* L. victoriae*. This is achieved by chemical manipulation of the odour profile of heterospecific females, so that males perceive them as conspecifics and show antennal courtship behaviour. Males courted the manipulated heterospecific females and antennal contact between the male and the female was observed. However, males elicited receptiveness only in conspecific females, never in the manipulated heterospecific females. Chemical analysis showed the presence of species specific unsaturated hydrocarbons on the antennae of males. Only trace amounts of these hydrocarbons are found on the antennae of females. Our results are an important step towards the understanding and identification of antennal pheromones of parasitic wasps.

## 1. Introduction

Chemical senses are widespread in nature and chemical communication was very likely the first mechanism to transfer information between individuals [1]. Chemical compounds that transfer information can be divided into allelochemicals and pheromones [2]. Wyatt [3] defines “pheromones” as “molecules that are evolved signals which elicit a specific reaction, for example, a stereotyped behaviour and/or a developmental process in a conspecific.” Sex pheromones are thus signals that are involved in behaviour or processes that relate to mating.

In parasitic Hymenoptera, sex pheromones are important at three different levels of sexual communication [4]:

1. Mate attraction: one sex attracts the other over some distance with a volatile pheromone.
2. Mate recognition: less volatile pheromones facilitate reliable recognition of sex and species to identify a specimen as a suitable mate and elicit courtship.
3. Courtship: during courtship, males release aphrodisiac pheromones to elicit female receptiveness.

Aphrodisiac pheromones are often employed by males to elicit receptiveness in females and their involvement in courtship has been extensively investigated in parasitic Hymenoptera. In several species, including* Leptopilina*, antennal or oral male aphrodisiac pheromones have been proposed [5–10]. Such male aphrodisiac pheromones could allow females to identify a courting male as conspecific, if the pheromone is species specific. Additionally, an aphrodisiac pheromone could also signal male quality [11]. In some species, for example, in the genus* Nasonia*, the male aphrodisiac pheromone lacks species specificity, as heterospecific courting males may be accepted as a mate by a female.* Nasonia* species also possess very similar sex pheromones, which leads to interspecific courtship [12]. In other genera, such as* Leptopilina*, mate recognition is species specific [13, 14], which usually prevents interspecific courtship.

Isidoro et al. [8] proposed that a male antennal aphrodisiac pheromone also exists in* L. boulardi.* In their work, they demonstrated that antennal contact between males and females during courtship is required to elicit receptiveness in females. Additionally, they described glands and gland openings in the third and fourth male antennomeres. These antennomeres are brought into contact with the distal part of the female antennae during courtship [8]. It is thus assumed that a chemical substance, an aphrodisiac pheromone, is transferred from the male antennae onto the female antennae to elicit female receptiveness. The species specificity of the proposed aphrodisiac pheromone, however, could not be investigated in* L. boulardi* and* L. heterotoma,* as interspecific courtship rarely occurs. Using chemical manipulation of females, we investigated the species specificity of the male courtship signals (putatively pheromones) in* L. heterotoma, L. boulardi,* and* Leptopilina victoriae*. Additionally we analysed the chemical compounds found on the antennae of the males of these three species. Our results are an important step towards the understanding and identification of antennal pheromones on parasitic wasps.

## 2. Material and Methods

### 2.1. Insects

We reared* L. boulardi, L. heterotoma,* and* L. victoriae* using* Drosophila melanogaster* as the host species.* Drosophila melanogaster* was reared on a corn-based diet (504 mL water, 66 g sugar, 6 g baker's yeast, 2.3 g agar, 52 g cornmeal, 1.3 mL propanoic acid, and 0.8 g Nipagin) at 25°C ambient temperature, with roughly 75% humidity, and a 16 : 8 h L : D cycle. About 30 flies (mixed sexes) were placed into a jar for each rearing. The jar contained fresh fly food. The flies were removed from the jar after 48 h, and about 10 wasps (both sexes) were put into the jar. Parasitized fly pupae were removed from the jars before the adult wasps emerged and put singly into 1.5 mL microcentrifuge tubes to obtain naive and virgin wasps of known age.

### 2.2. Extraction

Virgin 1-day-old females were extracted in batches of 30–50 with 5 *μ*L dichloromethane (DCM) per female for 10 min. Afterwards, the DCM was evaporated under a gentle stream of nitrogen. Then, the residue was redissolved in 1 *μ*L acetone per 5 *μ*L original volume. The final concentration of the extract thus equalled 1 female per 1 *μ*L.

For the analysis of the antennae we killed 1-2-day-old naive males by freezing them. We then cut off the antennae and extracted them in batches of 10 (=5 males) in 25 *μ*L DCM. The body of the males was also extracted in 50 *μ*L DCM. We analysed 10 to 12 samples per species.

### 2.3. Chemical Analysis

Extracts and fractions were analysed on a GC2010 gas chromatograph (GC, Shimadzu, Duisburg, Germany) connected to a QP2010 plus mass spectrometer (MS, Shimadzu, Duisburg, Germany). The GC was equipped with a nonpolar capillary column (BPX-5, 30 m length, 0.25 mm inner diameter, and 0.25 *μ*m film thickness, SGE Analytical Sciences, Milton Keynes, UK). Helium was used as carrier gas with a constant linear velocity of 50 cm s^−1^. The temperature of the GC oven started at 80°C and was raised by 5°C min^−1^ to 280°C, where it was kept for 20 min. The MS was run in electron impact (EI) mode at 70 eV and set to a scan range from 35 to 600 mz^−1^. Sample volumes of 1 *μ*L were injected splitless at an injector temperature of 280°C. The n-alkanes in the extracts of males of all three* Leptopilina* species were identified by comparing mass spectra and retention indices to those of synthetic reference compounds. Methyl-branched hydrocarbons were identified by interpretation of diagnostic ions resulting from the favoured fragmentation at the branching points [15] and comparison of linear retention indices with literature data [16].

### 2.4. Mating Trials

To investigate whether males from each species could elicit readiness to mate with con- and heterospecific females, mating trials were conducted. For this, naive, virgin males were allowed to court naive, virgin females and we recorded whether females showed readiness to mate. Trials were conducted in a small plexiglass arena (15 mm diameter and 2 mm height) covered with a glass lid and lasted 120 s. Trials were terminated early when the female showed readiness to mate. As males court only conspecific females, heterospecific female odour profiles had to be chemically manipulated, so the females were perceived as conspecifics by the males. The female odour profiles were manipulated by applying 0.1 *μ*L (equalling 0.1 female equivalents) female extract redissolved in acetone from the male's species to the female. Previous studies [17, 18] have revealed that many parasitic wasps tolerate the application of acetone extracts without any visible intoxication. Conspecific females were also treated with extract. The extract was applied using on-column GC syringe (Hamilton, Bonaduz, Switzerland). After applying the extract, females were allowed to recover for 120 s. Females that did not recover after the application were discarded from the experiment (only 2 of all treated females did not recover within 120 s). After recovering, females were carefully placed into the arena and a single male was added. We recorded whether the male courted the female and whether the female showed readiness to mate. For each possible combination of male and female species, we conducted experiments until male courtship including antennal stroking was observed in 10 replicates.

### 2.5. Statistical Analysis

The number of mating trials conducted until courtship including antennal stroking was observed 10 times in each combination of male and female species and was analysed with the chi-squared test. The relative amounts of the compounds found in the extracts were arc-sinus transformed before statistical analysis. To compare the chemical profiles of the three species we performed a nonparametric multidimensional scaling based on the Bray-Curtis distance between samples. Statistical tests were performed in Past 3 [19] and R version 3.1.1 [20].

## 3. Results

**Figure 1:**
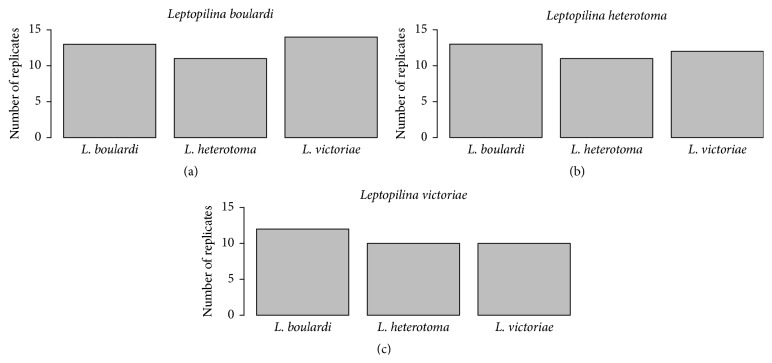
Number of replicates conducted until courtship including antennal stroking was observed 10 times in intraspecific and interspecific mating trials. A statistical analysis of the number of conducted replicates showed no significant differences between the different species combinations (chi-squared test: *χ* ^2^ = 0.2527; df = 4; *p* = 0.9927).

**Figure 2:**
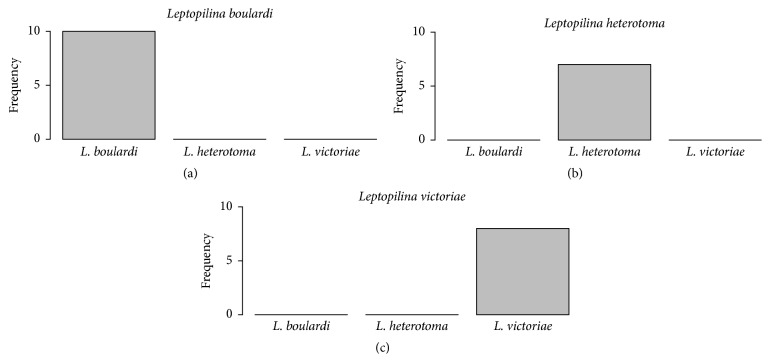
Number of (a)* L. boulardi* males, (b)* L. heterotoma* males, and (c)* L. victoriae* males that elicited readiness to mate in mating trials with conspecific and heterospecific females. For each experiment *n* = 10.

Males of all three species readily courted both conspecific females and heterospecific females treated with extract of conspecific females (Figure 1). The statistical analysis of the number of conducted replicates indicated no significant differences between all combinations of male and female species (chi-squared test: *χ* ^2^ = 0.2527; df = 4; *p* = 0.9927). The manipulation of the females' odour profiles was an effective means to reliably elicit interspecific courtship. However, males elicited readiness to mate only with conspecific females but never with heterospecific females (Figure 2).

**Figure 3:**
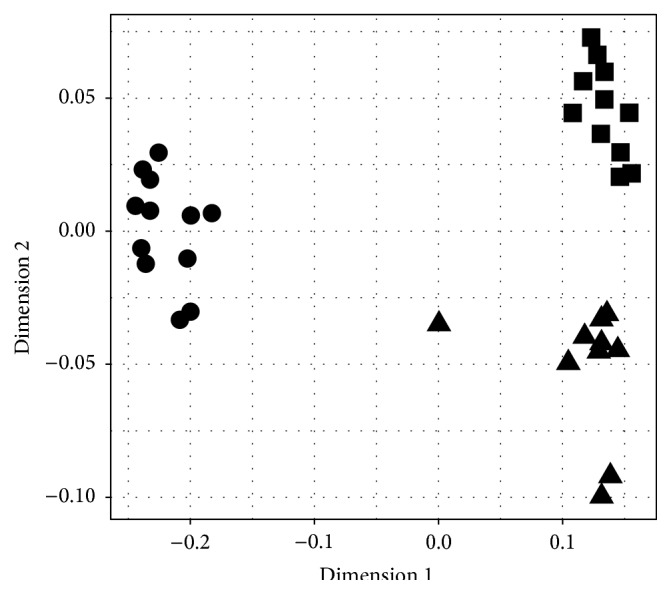
Nonparametric multidimensional scaling (NMDS) analysis of the chemical profiles on the antennae of males of* L. heterotoma* (triangles),* L. boulardi* (circles), and* L. victoriae* (squares).

We found 24 compounds in the extracts of the antennae of males (Table 1). The chemical profiles of males of* L. heterotoma* and* L. victoriae* were dominated by pentatriacontadiene (C35), while males of* L. boulardi* produce high amounts of two methyl-branched alkenes with a chain length of 31. Those unsaturated hydrocarbons are only present in trace amounts in the chemical profiles of females [14]. The chemical compounds found on the antennae of males were also found on the other parts of the body of males (not shown). The statistical analysis (NMDS stress = 0.12) showed that the chemical profiles of the antennae of males are species specific (Figure 3).

## 4. Discussion

We have found that the male courtship signal (putatively an antennal aphrodisiac pheromone) in* Leptopilina* is species specific. Males of the species* L. heterotoma, L. boulardi,* and* L. victoriae* can elicit readiness to mate only with conspecific, but not in heterospecific females.

Isidoro et al. [8] demonstrated that antennal contact during courtship is essential in* L. boulardi*. They showed elegantly by amputation of antennae that males cannot elicit readiness to mate with females if antennal contact is prevented. In their experiments, they used individuals that had one of their antennae amputated. When females and males had the antennae amputated on the same side, males were still able to elicit receptiveness in the females, but when females and males had the antennae amputated on different sides, males failed to elicit receptiveness. However, Isidoro et al. [8] could not investigate the species specificity of the assumed male antennal aphrodisiac pheromone, as* L. heterotoma* males did not court* L. boulardi* females—and vice versa—in the bioassays. The absence of cross-specific courtship is no surprise, as mate recognition is species specific in* Leptopilina* [14]. We could overcome this problem by chemically manipulating the odour profile of heterospecific females, so males perceived them as conspecifics. Males of the three investigated species readily courted these manipulated females, which allowed us to investigate the species specificity of the male antennal pheromone.

One possible explanation for the required antennal contact demonstrated by Isidoro et al. [8] is that the signal is a tactile one. Males from different species could show different stroking patterns and, for example, the stroking speed could signal mate quality. Such signalling is known from, for example, cucumber beetles, in which the female decides to reject or accept the male's spermatophore based on antennal stroking speed [21]. In the mating trials conducted in the present study, however, no obvious species specific antennal stroking patterns were observed.

Antennal glands in males, on the other hand, are a common feature in Hymenoptera [10], and they are putatively involved in courtship in a number of parasitic Hymenoptera. Strong evidence for male antennal aphrodisiac pheromones has been found in, for example,* Amitus spiniferus* (Hymenoptera: Platygastridae) [6],* Pimpla turionellae* (Hymenoptera: Ichneumonidae) [7],* Trichopria drosophilae* (Hymenoptera: Diapriidae) [10], and also* L. boulardi* [8]. We thus assume that the species specific signal in* Leptopilina* is indeed a pheromone.

This assumption is supported by the results of the chemical analysis. Males of all three species produce double unsaturated (*L. heterotoma* and* L. victoriae*) or methyl-branched monounsaturated hydrocarbons (*L. boulardi*) with a chain length of 31 to 35. These compounds dominate the cuticular hydrocarbon profiles of males but are only found in trace amounts in females. For example, in* L. heterotoma*, the diene accounts for more than 45% of the total amount of hydrocarbons found on the male antennae (Table 1). The chemical profiles found on the male antennae are also species specific (Figure 3) and are thus ideal compounds to form the putative male aphrodisiac pheromone. The very low volatility of the compounds fits well to the direct contact of the antennae needed to elicit receptiveness in the females. We found the male specific compounds also on the other parts of the males' body, not only on the antennae. The grooming behaviour regularly shown by the males (personal observation) could account for an even spread of the compounds over the whole body of the males.

We found that the male courtship signal (putatively a pheromone) is indeed species specific. This is noteworthy, as a species specific courtship signal establishes a barrier to heterospecific matings, even though mate recognition is already species specific [14]. There are thus two independent recognition mechanisms: the female pheromone eliciting courtship in the male and the male courtship signal eliciting receptiveness in the females. This might reflect a high selective pressure against heterospecific matings.* Leptopilina* females are, like most (solitary) parasitic Hymenoptera, monandrous ([22], personal observation for* L. heterotoma*). In combination with the arrhenotoky found in* Leptopilina* and the* Wolbachia*-mediated cytoplasmic incompatibility [23, 24], this imposes a great fitness loss upon females that accept a heterospecific mate. Only male offspring will be produced from heterospecific matings, which results in a reduced fitness as compared to producing female and male offspring. This is especially true for species that experience local mate competition, which is true for at least* L. heterotoma* [25].

Despite the mentioned range of literature showing the great interest in such male aphrodisiac pheromones in parasitic Hymenoptera, no such putative pheromone has been identified to date. Our experimental setup allows us to have males courting heterospecific females, in which they cannot elicit readiness to mate. In subsequent experiments, the identified pheromone candidate compounds should now be applied to male antennae in heterospecific courtship trials to investigate their ability to elicit female receptiveness.

## 5. Conclusions

In this study we could show that the male courtship signal of* Leptopilina* males is species specific. We assume that this courtship signal is a male antennal aphrodisiac pheromone and we found species specific long-chained hydrocarbons on the antennae of males which seem well suited to form these putative pheromones. The results are an important step towards the first identification of a male antennal pheromone in a parasitoid wasp.

## Figures and Tables

**Table 1:** Mean (±SD) relative amounts and linear retention indices of the compounds extracted from the antennae of males of *L. heterotoma, L. boulardi,* and *L. victoriae*.

Compound	Retention index	*L. heterotoma*		*L. boulardi*		*L. victoriae*	
		Mean	SD	Mean	SD	Mean	SD
4-Methyl octacosane	2858	10.95	2.47	21.59	3.40	6.97	1.02
Unidentified	2944	0.53	0.24	0.69	1.05	0.26	0.20
Methyl triacontane^1^	3024	0.08	0.14	3.81	0.99	0.27	0.09
4-Methyl triacontane	3056	16.70	4.93	10.69	1.25	26.79	6.78
Methyl hentriacontene^1^	3106	0.20	0.33	14.04	1.22	0.05	0.03
Methyl hentriacontene^1^	3114	0.16	0.30	11.82	2.22	0.31	0.17
Methyl hentriacontane^1^	3121	1.37	0.92	1.15	0.25	2.30	0.99
Unidentified	3140	4.42	1.59	7.96	1.18	5.09	1.24
Methyl hentriacontane^1^	3157	0.53	0.43	0.15	0.11	0.44	0.15
Methyl hentriacontane^1^	3168	0.59	0.46	0.22	0.10	0.82	0.39
Methyl dotriacontane^1^	3222	0.24	0.20	1.38	0.33	0.61	0.31
Tritriacontadiene^1^	3242	2.83	1.12	15.29	4.93	2.07	0.56
Tritriacontadiene^1^	3249	2.24	0.89	0.03	0.04	3.26	1.26
4-Methyl dotriacontane	3255	3.59	1.03	0.27	0.14	3.92	1.14
Methyl tritriacontene^1^	3305	0.17	0.38	9.05	2.38	0.02	0.02
Methyl tritriacontene^1^	3314	1.73	1.57	0.23	0.08	0.32	0.18
Methyl tritriacontane^1^	3320	1.57	1.63	0.10	0.08	0.78	0.36
Unidentified	3336	0.95	0.37	1.14	1.71	0.00	0.00
Unidentified	3341	1.00	0.58	0.01	0.01	0.00	0.00
Unidentified	3342	0.00	0.00	0.00	0.00	0.96	0.50
Methyl tritriacontane^1^	3349	0.61	0.72	0.10	0.20	0.00	0.01
Methyl tritriacontane^1^	3355	0.98	0.96	0.02	0.05	0.00	0.00
Pentatriacontadiene^1^	3441	48.57	8.27	0.27	0.20	42.04	7.33
Pentatriacontadiene^1^	3448	0.00	0.00	0.00	0.00	2.72	0.85

^1^The position of the methyl branch and/or the double bond could not be determined.
